# Transluminal opening technique for multiple septa in liver abscess using novel cholangioscope

**DOI:** 10.1055/a-2371-1258

**Published:** 2024-08-07

**Authors:** Takeshi Ogura, Yuki Uba, Nobuhiro Hattori, Kimi Bessho, Hiroki Nishikawa

**Affiliations:** 1 Endoscopy Center, Osaka Medical and Pharmaceutical University Hospital, Osaka, Japan; 2 2nd Department of Internal Medicine, Osaka Medical and Pharmaceutical University, Osaka, Japan

The gold standard technique for liver abscess drainage is the percutaneous transhepatic approach 1 ; however, endoscopic ultrasound-guided liver abscess drainage (EUS-LAD) has been reported 2 3 4 5 to overcome several disadvantages of percutaneous transhepatic liver abscess drainage, including external drainage or the risk of self-tube removal. However, if the liver abscess has multiple septa, the drainage effect after EUS-LAD might be limited. Several techniques, such as guidewire manipulation, can be used to divide the septa. However, breaking the septum might be challenging in cases with thick-walled septa. In addition, if a blood vessel is present in the septal wall, the procedure carries the risk of bleeding.

Recently, a novel cholangioscope (EyeMAX; Micro-Tech Co., Ltd, Nanjing, China), with a large working channel, has been developed. This scope has several benefits, such as allowing favorable visualization because of strong injection and aspiration functions due to the large working channel. Herein, we describe a novel technique, called the “transluminal opening technique,” for managing multiple septa in a liver abscess using the novel cholangioscope.

A 90-year-old man was admitted for the treatment of liver abscess. EUS imaging demonstrated a liver abscess with multiple septa ( Fig. 1 ). As the patient had dementia, a transluminal approach to the abscess was selected. After liver abscess puncture and contrast medium injection ( Fig. 2 ), a balloon catheter was inserted. Then, division of the septa was performed as much as possible ( Fig. 3 ). Finally, EUS-LAD using a metal stent was performed.

**Fig. 1:**
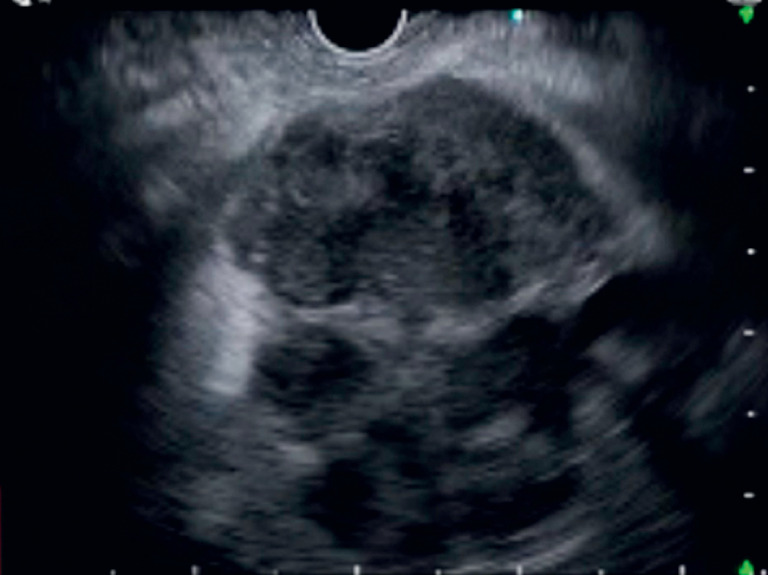
Multiple septa were observed within the liver abscess.

**Fig. 2:**
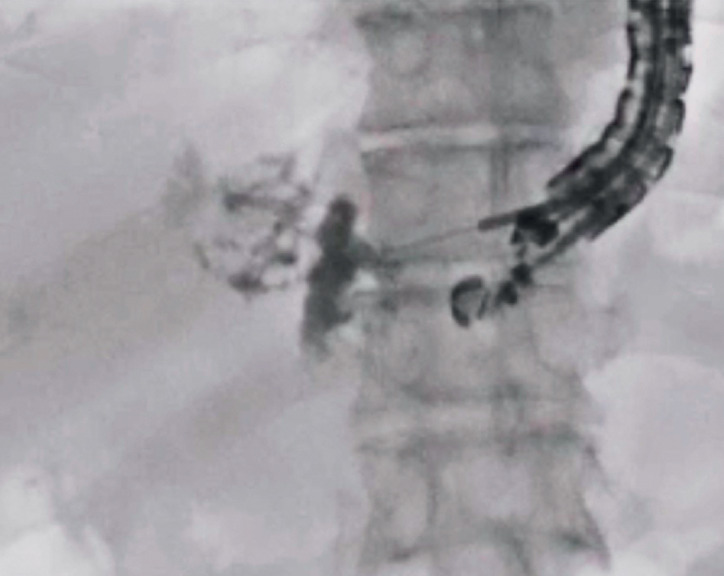
The liver abscess was punctured using a 19-G needle and contrast medium was injected.

**Fig. 3:**
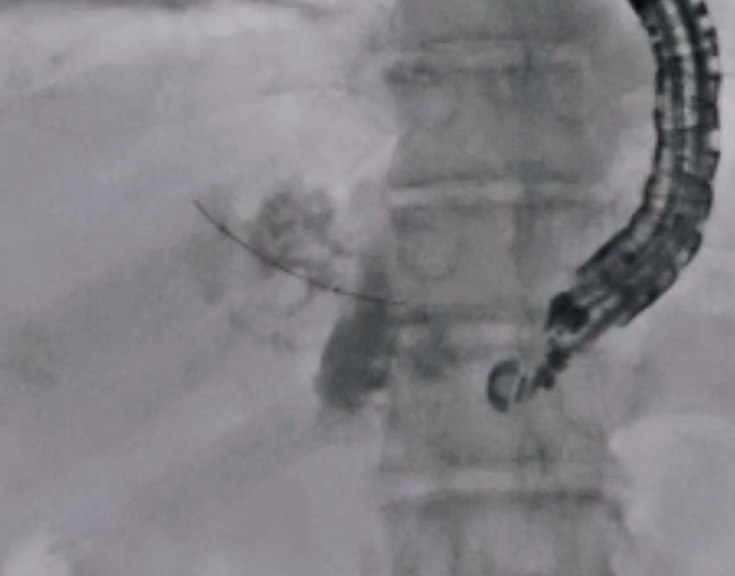
The septa were broken, as much as possible, using a catheter and guidewire.

Owing to inadequate clinical effects, a transluminal opening technique was attempted after 7 days. First, an endoscopic retrograde cholangiopancreatography catheter was inserted within the EUS-LAD stent, and the contrast medium was injected. However, the cavity of the liver abscess was small. To break the septa, the novel cholangioscope was inserted within the liver abscess via a fistula. The septa were broken using biopsy forceps under direct visualization ( Fig. 4 ), and the cavity of the liver abscess was opened. A double-pigtail plastic stent was deployed without any adverse events ( Fig. 5 , Video 1 ). Following this procedure, the patient’s clinical course was excellent and he was discharged after 10 days.

**Fig. 4:**
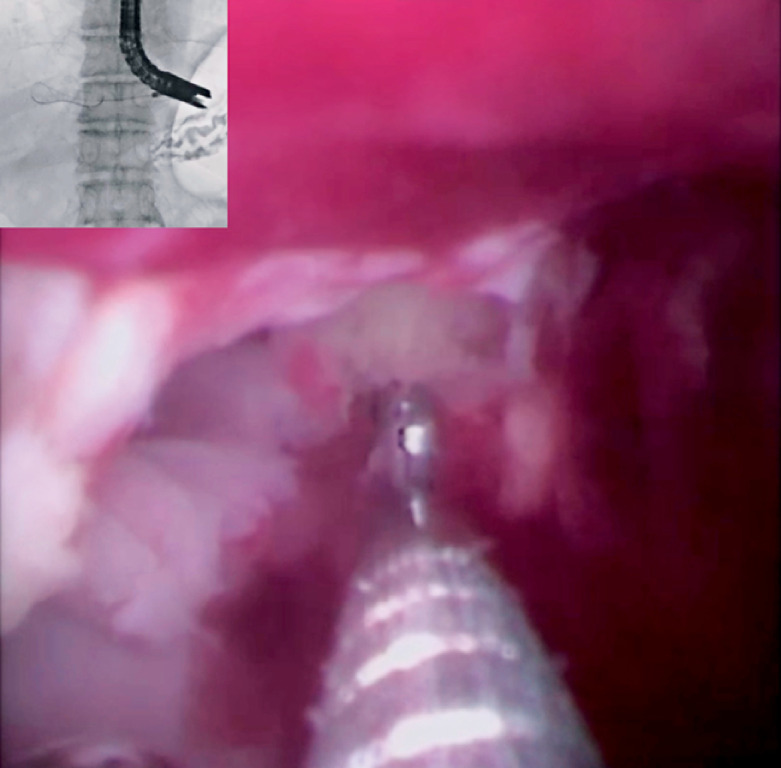
The septa were broken using biopsy forceps under cholangioscopic guidance.

**Fig. 5:**
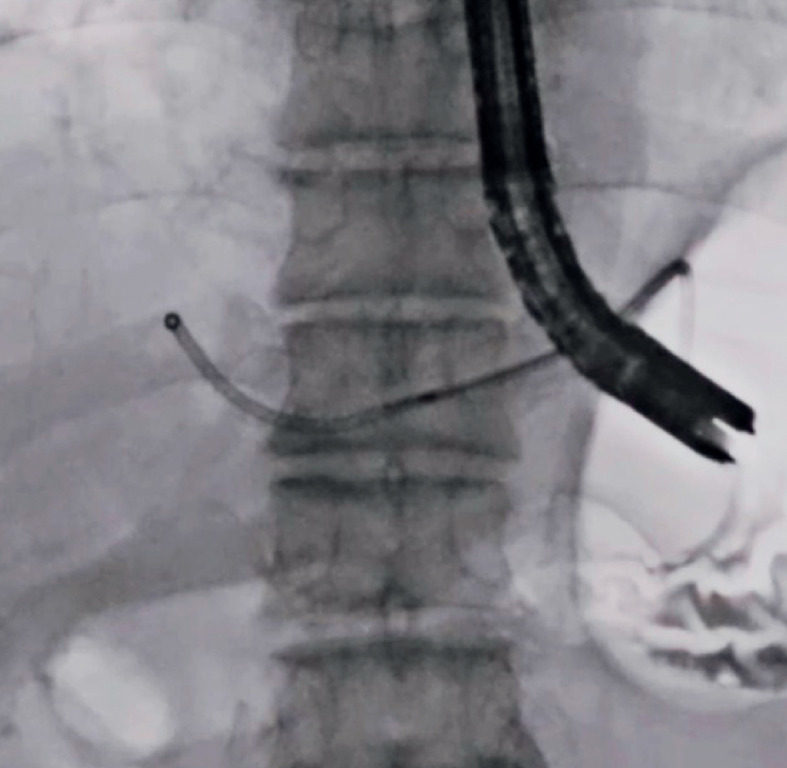
A plastic stent was deployed.

The septa were broken using biopsy forceps under cholangioscopic guidance.Video 1

In conclusion, the present technique might be useful for liver abscesses with multiple septa.

Endoscopy_UCTN_Code_TTT_1AS_2AG

## Notes

Endoscopy E-Videos https://eref.thieme.de/e-videos
*E-Videos* is an open access online section of the journal *Endoscopy* , reporting on interesting cases and new techniques in gastroenterological endoscopy. All papers include a high-quality video and are published with a Creative Commons CC-BY license. Endoscopy E-Videos qualify for HINARI discounts and waivers and eligibility is automatically checked during the submission process. We grant 100% waivers to articles whose corresponding authors are based in Group A countries and 50% waivers to those who are based in Group B countries as classified by Research4Life (see: https://www.research4life.org/access/eligibility/ ). This section has its own submission website at https://mc.manuscriptcentral.com/e-videos .
